# The Current Role of Surgery in the Treatment of Cardiac Metastases from Malignant Melanoma: an Educational Presentation

**DOI:** 10.21470/1678-9741-2020-0379

**Published:** 2021

**Authors:** Kyriakos Spiliopoulos, Peter Engels, Konstantina Kimpouri, Iraklis Floudas, Nikolaos S. Salemis, Franz-Xaver Schmid

**Affiliations:** 1 Department of Cardiac Surgery, Helios Klinikum Krefeld, Germany.; 2 Department of Thoracic and Cardiovascular Surgery, University of Thessaly, Larissa, Greece.; 3 Dermomedica, Dermatology Center, Larissa, Greece.; 4 Breast Cancer Surgery Unit, Army General Hospital, Athens, Greece.

**Keywords:** Melanoma, Skin Neoplasms Recurrence, Local, Fatigue, Dyspnea, Ventricular Outflow Obstruction, Shoulder

## Abstract

A 71 year-old male with a history of multiple excisions of an initial Clark's level V melanoma of the breast followed by combined radiation and interferon treatment, as well as a recurrence, 3 years later, of a BRAF-positive tumor of the shoulder, with subsequent therapy with dabrafenib and trametinib, presented again with progressive intracardiac masses causing significant right ventricular outflow obstruction. Additionally, the patient complained of dyspnea and fatigue on exertion, thus he was scheduled for surgical resection.

**Table:** 

Abbreviations, acronyms & symbols	
CT	= Computed tomography
PET	= Positron emission tomography
MRI	= Magnetic resonance imaging

## Questions

- What is the epidemiology of cardiac metastatic melanoma?
- What is the pathophysiology of cardiac metastatic melanoma?
- What is the most adequate imaging technique for the diagnosis?
- What are the insights, pitfalls, aims and impact of the surgical treatment?
- What is new in the melanoma treatment?
- Is there a therapeutic algorithm?

## DISCUSSION

Melanoma has the highest propensity for cardiac metastases among malignant tumors. In the largest autopsy series of metastatic melanoma reported, more than 60% of the cases showed cardiac involvement. On the contrary, metastases of the primary tumor are found only in less than 2% of patients *ante-mortem.* This discrepancy between *ante-* and *post-mortem* manifestations may be attributed to the variability or lack in clinical signs. Cardiac metastases are usually clinically silent and only 20-30% of the patients develop impaired cardiac function. Cardiac manifestations in general occur late in the course of the disease, when there is already a widespread dissemination of the tumor to several organs (Question A).

Cardiac metastatic melanoma occurs primarily via hematologic dissemination anywhere in the heart, invading the walls of all 4 cardiac chambers, with the right atrium being the most frequently affected. They mostly involve the pericardium and myocardium appearing with multifocal lesions, while the endocardium is rarely affected. Solitary metastases from melanoma in a cardiac chamber is uncommon, thus anecdotally reported. Especially when the tumor is localized in the left chambers, a possible patent foramen ovale should be ruled out through a transesophageal echocardiograph (Question B).

**Fig. 1:**
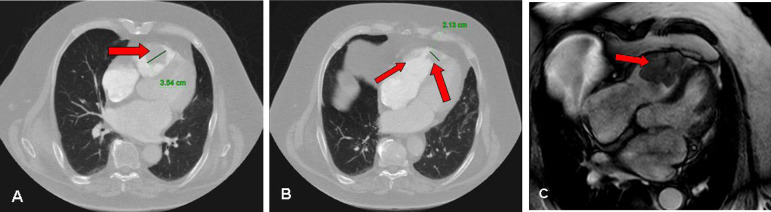
Radiological findings. (A, B) Contrast-enhanced axial computed tomography images through the heart showing tumor masses (red arrows) at the apex and anterior right ventricular wall of a measured diameter of 2.1-3.6 cm. (C) Magnetic resonance image obtained in long-axis projection through the right ventricle. The red arrow shows the mass filling the right ventricular cavity.

Currently, the gold standard for diagnostic imaging is positron emission tomography-computed tomography (PET-CT), which can be used concomitantly with magnetic resonance imaging (MRI) of the brain and CT of the chest, abdomen, and pelvis (Figure 1). One of the reasons why cardiac metastasis is so easily un- or underdiagnosed is that most patients remain free of specific cardiac symptoms. Of the imaging modalities employed to identify cardiac masses, cardiac-MRI has demonstrated the best utility (Question C).

Regarding the surgical techniques, these are determined by the anatomic site and extent of the tumor growth and anecdotally have even employed *ex-vivo* resection and autotransplantation. The aim of the surgical procedures should be a complete resection of all masses with tumor-free margins, when technically/anatomically feasible, followed by accurate anatomic reconstruction. Despite few reports presenting complete resection of metastatic melanoma from both atria and ventricles with survival up to one year, it is difficult to estimate the overall morbidity and mortality related to the resection of cardiac melanoma metastases, due to the lack of longitudinal data in the current literature (Question D).

Despite the dismal prognosis in standard chemotherapies with 5-year survival rates of 5-10%, the introduction of BRAF- and MEK-targeted therapies and immune checkpoint inhibitors in 2011 resulted in significant better outcomes. In a recent published pooled analysis of extended survival data from two trials (COMBI-d and COMBI-v) evaluating patients not previously treated with unresectable or metastatic melanoma and a BRAF V600E or V600K mutation, who had received BRAF inhibitor dabrafenib plus MEK inhibitor trametinib, the estimated overall survival and 5-year progression-free survival were 34% and 19%, respectively (Question E).

The management of cardiac metastatic melanoma should be individualized according to the characteristics of the patient and the tumor. In asymptomatic patients with solitary cardiac metastasis, if complete surgical resection to clean the margins seems feasible and removal would render them in a status of a non-evident disease, surgery is a reasonable first choice therapeutical option. On the other hand, in case of multiple and/or invading tumors, or further systemic disease dissemination, not amenable to complete resection, especially in cases positive for BRAF mutation, then BRAF, MEK, and immune checkpoint inhibitors should be considered as first-line therapy.

Regarding symptomatic patients, the following conditions are to be mentioned:

- In cases with solitary or disseminated symptomatic, but not acute life-threatening and BRAF mutation positive metastases, treatments with targeted inhibitors showed a high response rate with a short median response time. Subsequently, this may result either in regression of symptomatic lesions and/or in converting previously unresectable lesions into surgically removable ones.
- Highly symptomatic patients with hemodynamic failure should undergo urgent surgical complete resection with clear margins, if feasible (Question F).

### Brief Consideration of the Case Reported

**Fig. 2:**
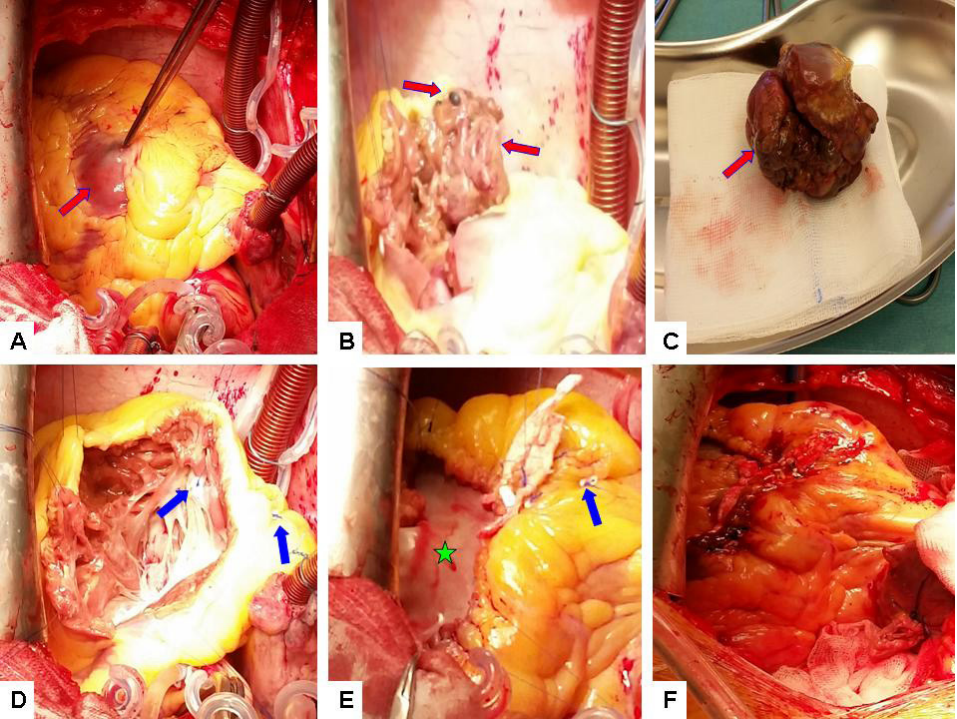
Surgical resection of cardiac metastatic melanoma (view from the patient's head). (A) Standard setting with aortic and bicaval cannulation, antegrade cardioplegia. Red arrow pointing at the right ventricular tumor mass. (B) Black tumor nodules in the endocardium (red arrows). (C) Macroscopic view of the tumorous mass excised from the right ventricle with an approximate size of 7 mm (red arrow). (D) Stabilization of the tricuspid valve with a fixation stitch (blue arrows). (E) Reconstruction of the resulting ventricular wall defect with bovine pericardial patch (green star), blue arrow pointing at the fixation stitch. (F) Final result after reconstruction and decannulation.

In the case presented, the indication for surgical intervention was justified, as there was no clear evidence of further spread of the disease, the patient had cardiac symptoms and there was an intention for treatment escalation with immunotherapy. Under cardiopulmonary bypass, the tumor in the right ventricle was approached through a vertical incision extending from the lower mid portion of the anterior wall to the outflow tract. In addition to the resection of multiple tumor masses from the right atrium, ventricle and outflow tract with sizes of approximately 15, 40 and 70 mm respectively, stabilization of the tricuspid valve was performed using a fixation stitch. The resulting ventricular wall defect was repaired with a bovine pericardial patch (Figure 2). The patient had an uneventful postoperative recovery. The postoperative echocardiograms performed showed good biventricular function, without obstruction of the outflow tract or valvular regurgitation. Pathology and immunochemistry confirmed the diagnosis of metastatic melanoma. He was discharged from the hospital in good physical condition on the 15^th^ postoperative day and was referred for further immunotherapy with PD-1-inhibitor pembrolizumab. Unfortunately, the patient died suddenly due to an unknown cause, shortly after receiving the first treatment cycle on the 42^nd^ postoperative day^[1-9]^.

### Learning Points

- Early diagnosis of cardiac involvement in patients suffering from malignant melanoma is crucial.
- The development and introduction of new systemic therapies and the timely appropriate integration of surgery in the therapeutic scheme may be beneficial to the outcome, especially in cases with isolated lesions.
- The prognosis for metastatic melanoma remains poor, enabling to surgery predominantly only a palliative role.

**Table:** 

Authors' roles & responsibilities	
KS	Substantial contributions to the conception or design of the work; or the acquisition, analysis or interpretation of data for the work; final approval of the version to be published
PE	Drafting the work or revising it critically for important intellectual content; final approval of the version to be published
KK	Drafting the work or revising it critically for important intellectual content; final approval of the version to be published
IF	Drafting the work or revising it critically for important intellectual content; final approval of the version to be published
NSS	NSS Drafting the work or revising it critically for important intellectual content; final approval of the version to be published
FXS	Drafting the work or revising it critically for important intellectual content; final approval of the version to be published
